# Laparoscopic Pancreaticoduodenectomy *Versus* Conventional Open Approach for Patients With Pancreatic Duct Adenocarcinoma: An Up-to-Date Systematic Review and Meta-Analysis

**DOI:** 10.3389/fonc.2021.749140

**Published:** 2021-10-27

**Authors:** Qingbo Feng, Wenwei Liao, Zechang Xin, Hongyu Jin, Jinpeng Du, Yunshi Cai, Mingheng Liao, Kefei Yuan, Yong Zeng

**Affiliations:** ^1^Department of Liver Surgery and Liver Transplantation Centre, West China Hospital, Sichuan University, Chengdu, China; ^2^Key Laboratory of Carcinogenesis and Translational Research (Ministry of Education/Beijing), Department of Hepatobiliary and Pancreatic Surgery Unit I, Peking University Cancer Hospital & Institute, Beijing, China

**Keywords:** pancreatic ductal adenocarcinoma, pancreaticoduodenectomy, laparoscopic, meta-analysis, whipple

## Abstract

**Background:**

To compare perioperative and oncological outcomes of pancreatic duct adenocarcinoma (PDAC) after laparoscopic *versus* open pancreaticoduodenectomy (LPD *vs.* OPD), we performed a meta-analysis of currently available propensity score matching studies and large-scale retrospective cohorts to compare the safety and overall effect of LPD to OPD for patients with PDAC.

**Methods:**

A meta-analysis was registered at PROSPERO and the registration number is CRD42021250395. PubMed, Web of Science, EMBASE, Cochrane Central Register of Controlled Trials, and ClinicalTrials.gov databases were searched based on a defined search strategy to identify eligible studies before March 2021. Data on operative times, blood loss, 30-day mortality, reoperation, length of hospital stay (LOS), overall morbidity, Clavien–Dindo ≥3 complications, postoperative pancreatic fistula (POPF), blood transfusion, delayed gastric emptying (DGE), postpancreatectomy hemorrhage (PPH), and oncologic outcomes (R0 resection, lymph node dissection, overall survival, and long-term survival) were subjected to meta-analysis.

**Results:**

Overall, we identified 10 retrospective studies enrolling a total of 11,535 patients (1,514 and 10,021 patients underwent LPD and OPD, respectively). The present meta-analysis showed that there were no significant differences in overall survival time, 1-year survival, 2-year survival, 30-day mortality, Clavien-Dindo ≥3 complications, POPF, DGE, PPH, and lymph node dissection between the LPD and OPD groups. Nevertheless, compared with the OPD group, LPD resulted in significantly higher rate of R0 resection (OR: 1.22; 95% CI 1.06–1.40; *p* = 0.005), longer operative time (WMD: 60.01 min; 95% CI 23.23–96.79; *p* = 0.001), lower Clavien–Dindo grade ≥III rate (*p* = 0.02), less blood loss (WMD: −96.49 ml; 95% CI −165.14 to −27.83; *p* = 0.006), lower overall morbidity rate (OR: 0.65; 95% CI 0.50 to 0.85; *p* = 0.002), shorter LOS (MD = −2.73; 95% CI −4.44 to −1.03; *p* = 0.002), higher 4-year survival time (*p* = 0.04), 5-year survival time (*p* = 0.001), and earlier time to starting adjuvant chemotherapy after surgery (OR: −10.86; 95% CI −19.42 to −2.30; *p* = 0.01).

**Conclusions:**

LPD is a safe and feasible alternative to OPD for patients with PDAC, and compared with OPD, LPD seemed to provide a similar OS.

**Systematic Review Registration:**

https://www.crd.york.ac.uk/PROSPERO/#recordDetails.

## Introduction

The incidence of pancreatic cancer (PC) has risen, and PC is likely to become the second most frequent cause of cancer-related death by 2030 (1). Moreover, pancreatic duct adenocarcinoma (PDAC) is the most common type of PC and the 14th most common cancer all over the world (2). Despite significant progress in adjuvant and neoadjuvant treatment options, pancreaticoduodenectomy (PD) has been shown to be the only effective and potentially curative treatment which can provide cure or prolonged survival for patients diagnosed to have PDAC. Due to the complexity of PD, it is often performed by laparotomy. But since Gagner and Pomp reported laparoscopic pancreaticoduodenectomy (LPD) in 1994 (3), LPD is increasingly used worldwide. Previous literature has confirmed the safety and feasibility of LPD and emphasized that it is superior to open pancreaticoduodenectomy (OPD) in reducing blood loss and involves shorter hospital stay, earlier oral intake, less pain, and faster recovery (4–6). Despite that there have been several other published meta-analyses assessing surgical and oncological outcomes between LPD and OPD in the past 3 years (7–9), the results of these studies are controversial. For this reason, which one is the better approach for PDAC, LPD or OPD, is unclear. We carried out an up-to-date meta-analysis to specifically study the perioperative, oncological outcomes and long-term survival of LPD *versus* OPD for PDAC.

## Methods

### Search Strategy and Study Selection

This study followed the PRISMA guidelines (10). Published studies which investigated LPD *versus* OPD for PDCA were systematically searched in PubMed, Web of Science, EMBASE, Cochrane Central Register of Controlled Trials, and ClinicalTrials.gov databases before March 22, 2021, by two independent researchers (QF, WL). The combinations of the following key terms and their combinations were used: laparoscopic, open, conventional, whipple, and pancreaticoduodenectomy. In order to gain additional studies, the references of eligible studies were manually searched.

### Inclusion and Exclusion Criteria

Two investigators (QF, WL) reviewed currently available literature and screened all titles and abstracts independently and identified eligible studies according to the following criteria.

The inclusion criteria were as follows: 1) participants: patients with PDAC; 2) types of interventions: LPD and OPD; 3) types of studies: randomized controlled trials (RCTs), retrospective studies, cohort studies, and case–control studies; and 4) primary articles published in English.

The exclusion criteria were as follows: 1) non-English or experimental studies; 2) studies without available data; and 3) the publication type was editorial, abstract, letter, case report, and expert opinion.

### Data Extraction and Quality Assessment

The original data from all candidate articles were independently assessed and extracted by two reviewers (QF, WL) by using a unified datasheet, and any ambiguity was resolved by a third researcher (ZX) who was consulted to review the study to reach a consensus. Data extracted included the following items: study and patient characteristics and operative and postoperative outcomes. Study and patient characteristics include first author, country, publication year, research design, sample size, and mean age; the latter included operative time, blood loss, blood transfusion, overall morbidity and 30-day mortality, length of hospital stay (LOS), R0 resections, number of harvested lymph nodes, overall survival (OS), postoperative long-term survival, and the time to starting postsurgical adjuvant chemotherapy. The quality of the included studies was assessed by the Newcastle-Ottawa Scale (NOS) (11). Every included study was independently evaluated by two authors (QF, WL), and NOS score ≥6 is considered as being of high quality.

### Statistical Analysis

Review Manager 5.3 software was used to analyze the data. The weighted mean difference (WMD) with the 95% confidence interval (CI) and odds ratio (OR) were used to compare continuous variables and dichotomous, respectively. For overall survival data, the method of Tierney et al. was adopted to estimate natural logarithm hazard ratios (HRs), standard errors (SE), and 95% CIs from survival curves (12). We adopted the method described by Hozo et al. to convert medians with ranges into means with standard deviations (13). Begg’s funnel plot and Egger’s test were used to assess potential publication bias. Statistical heterogeneity was quantified using Higgin’s *I*^2^ index. A fixed-effects model (FEM) was adopted when heterogeneity is low or moderate (*I*^2^ < 50%), while when heterogeneity is high (*I*^2^ ≥ 50%), a random-effects model (REM) will be used.

## Results

### Search Results and Characteristics of the Included Studies

**Figure 1:**
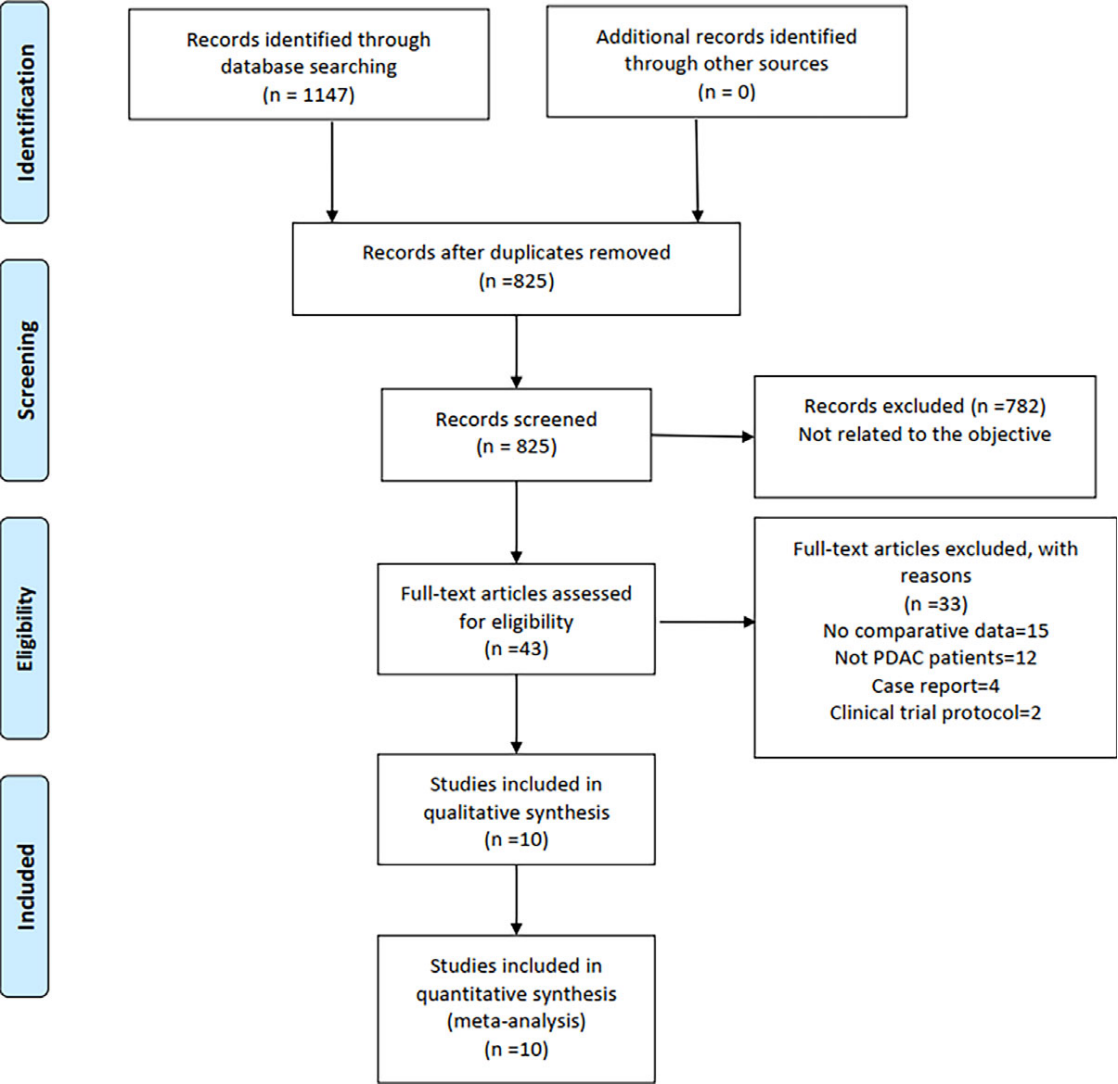
Flowchart of study identification and selection.

A total of 1,147 relevant English publications from various electronic databases were obtained. Finally, according to the inclusion criteria, 10 retrospective studies (14–23) comparing LPD and OPD in a total of 11,535 patients (1,154 and 10,021 underwent LPD and OPD, respectively) were included for further analysis. A flow diagram of our analysis protocol is shown in **Figure 1**. The general information and NOS scores of all the included studies are listed in **Table 1**, while all results of this meta-analysis are presented in **Table 2**.

**Table 1:** The main characteristics and NOS scores of the included studies in this meta-analysis.

Study	Type of study	Research time	Country	Patients (LPD *vs.* OPD)		Age (years)		Gender (M/F)		BMI		NOS
				LPD	OPD	LPD	OPD	LPD	OPD	LPD	OPD	
Croome et al. (14)	Retrospective	2008–2013	USA	108	214	66.6 ± 9.6	65.4 ± 10.9	51/57	131/83	27.4 ± 5.4	27.2 ± 5.3	8
Song et al. (15)	Retrospective	2011–2014	South Korea	11	261	NA	NA	NA	NA	NA	NA	7
Dokmak et al. (16)	Retrospective	2007–2012	France	15	14	68.1 ± 7	61.8 ± 10.5	NA	NA	NA	NA	7
Delitto et al. (17)	Retrospective	2010–2014	USA	28	22	NA	NA	NA	NA	NA	NA	7
Stauffer et al. (18)	Retrospective	1995–2014	USA	58	193	66.3 ± 9.5	64.5 ± 19.8	32/26	96/97	25.9 (17.7–49.7)	25.6 (15.0–46.2)	8
Kantor et al. (19)	Retrospective	2010–2013	USA	828	7325	65.9 ± 10.7	65.7 ± 10.4	NA	NA	NA	NA	8
Kuesters et al. (20)	Retrospective	2010–2016	Germany	62	278	71	68	31/31	137/141	24.7 (15–39)	24.7 (16–46)	7
Chapman et al. (21)	Retrospective	2016–2017	Korea	248	1520	79.6	79.5	18/40	42/49	23.1 ± 2.5	24.5 ± 3.6	8
Zhou et al. (22)	Retrospective	2013–2017	China	55	93	63.0 (54.0–69.0)	64.0 (59.0–70.5)	40/15	68/25	23.0 (20.7–25.2)	23.0 (20.7–25.2)	8
Chen et al. (23)	Retrospective	2004–2020	China	101	101	62.4 ± 8.2	62.2 ± 8.4	67/34	67/34	22.3 ± 2.5	22.3 ± 2.5	8

LPD, laparoscopic pancreaticoduodenectomy; OPD, open pancreaticoduodenectomy; M/F, male/female; SD, standard deviation; BMI, body mass index; NA, not applicable.

**Table 2:** Summary results of the meta-analyses.

Outcomes of interest	Studies, *n*	LPD	OPD	WMD (95% CI)/OR (95% CI)	*p*-value	Heterogeneity			
						*χ* ^2^	*df*	*I*^2^,%	*p*-value
**Operative outcomes**									
Operative time (min)	5	384	879	60.01 (23.23, 96.79)	0.001	72.54	4	94	<0.00001
Blood loss (ml)	5	333	862	−96.49 (−165.14, −27.83)	0.006	22.42	4	82	0.0002
Blood transfusion	5	384	879	0.75 (0.36, 1.54)	0.43	20.71	4	81	0.0004
**Postoperative outcomes**									
Overall complication rates	5	344	800	0.65 (0.5, 0.85)	0.002	5.3	4	24	0.26
Clavien–Dindo grade ≥III	4	322	601	0.61 (0.4, 0.91)	0.02	1.49	3	0	0.69
30-day mortality	6	1,272	9,612	1.02 (0.72, 1.44)	0.93	2.93	4	0	0.57
Postoperative pancreatic fistula	5	337	615	0.75 (0.51, 1.11)	0.15	1.21	4	0	0.88
Delayed gastric emptying	5	337	615	0.52 (0.21, 1.28)	0.16	13.93	4	71	0.008
Post pancreatectomy hemorrhage	5	337	615	1.04 (0.6, 1.79)	0.9	1.48	4	0	0.83
Length of stay (days)	8	1,475	9,738	−2.73 (−4.44, −1.03)	0.002	79.42	7	91	<0.00001
**Short-term oncological outcomes**									
R0 resection rate	9	1,486	9,999	1.22 (1.06, 1.40)	0.005	8.53	8	6	0.38
Lymph node dissection	8	1,234	8,452	2.14 (−0.84, 5.12)	0.16	199.59	7	96	<0.00001
**Long-term oncological outcomes**									
Overall survival	8			0.99 (0.95, 1.02)	0.41	7.36	7	5	0.39
1-year survival	5	267	968	1.2 (0.87, 1.65)	0.28	2.47	4	0	0.65
2-year survival	5	267	968	1.25 (0.94, 1.66)	0.13	0.93	4	0	0.92
3-year survival	5	267	968	1.5 (1.12, 2.02)	0.007	6.76	4	41	0.15
4-year survival	4	239	946	1.73 (1.02, 2.93)	0.04	6.34	3	53	0.1
5-year survival	3	131	732	2.11 (1.35, 3.31)	0.001	1.72	2	0	0.42
Time to starting adjuvant chemotherapy after surgery	6	1,398	9,446	−10.86 (−19.42, −2.3)	0.01	166.78	5	97	<0.00001

LPD, laparoscopic pancreaticoduodenectomy; OPD, open pancreaticoduodenectomy; WMD, weighted mean difference; OR, odds ratio; CI, confidence interval.

### Perioperative Outcomes

#### Operative Time

**Figure 2:**
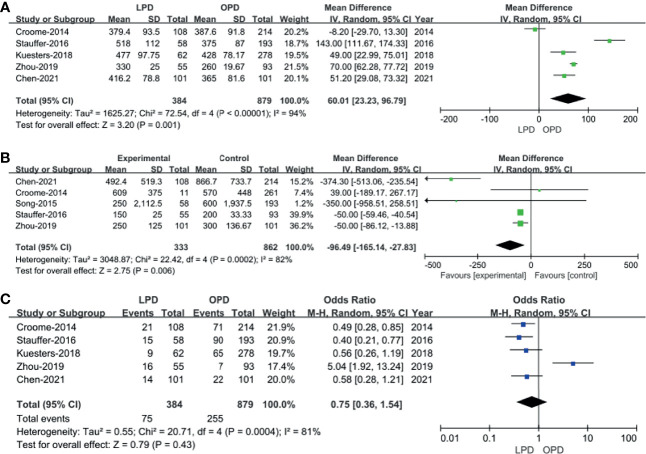
Forest plot of the comparison of laparoscopic pancreaticoduodenectomy (LPD) *versus* open pancreaticoduodenectomy (OPD) for operative outcomes. **(A)** Forest plot for operative time; **(B)** forest plot for blood loss; **(C)** forest plot for blood transfusion.

Five of the 10 included studies (14, 18, 20, 22, 23) that encompassed 1,263 patients (384 and 8,879 underwent LPD and OPD, respectively) reported operative times. We found that operative time was longer in the LPD group (WMD: 60.01 min; 95% CI 23.23–96.79; *p* = 0.001). Heterogeneity was high (*I*^2^ = 94%) and analyzed in REM (**Figure 2A**).

#### Blood Loss

Five studies with a total of 1,195 patients (14, 15, 18, 22, 23) had reported blood loss. The pooled data revealed that blood loss was lesser in the LPD group (WMD: −96.49 ml; 95% CI −165.14 to −27.83; *p* = 0.006). Heterogeneity was high (*I*^2^ = 82%) and analyzed in REM (**Figure 2B**).

#### Blood Transfusion

Blood transfusion rate data were available in five studies (14, 18, 20, 22, 23). The meta-analysis suggested that blood transfusion rate was not different in the two groups (OR: 0.75; 95% CI 0.36 to 1.54; *p* = 0.43). Heterogeneity was high (*I*^2^ = 81%) and analyzed in REM (**Figure 2C**).

### Postoperative Outcomes

#### Overall Complication Rates

**Figure 3:**
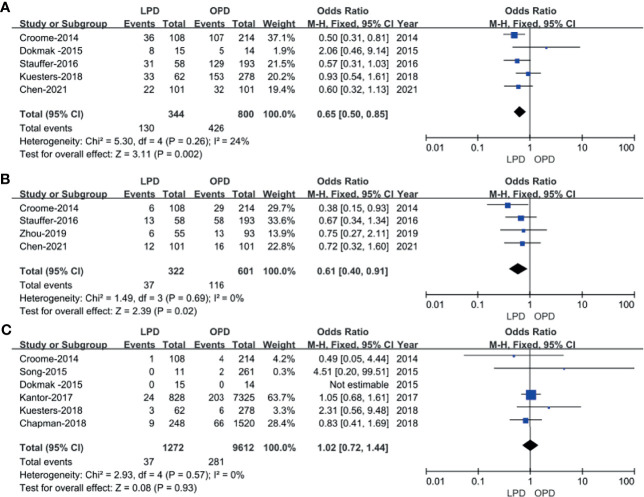
Forest plot of the comparison of LPD *versus* OPD for postoperative outcomes. **(A)** Forest plot for overall complication rates; **(B)** forest plot for Clavien–Dindo grade ≥III; **(C)** forest plot for 30-day mortality.

Five studies (14, 16, 18, 20, 23) that encompassed 1,144 patients (344 and 800 underwent LPD and OPD, respectively) recorded the postoperative complications, and the present analysis revealed lower overall complication rate in the LPD group (OR: 0.65; 95% CI 0.50 to 0.85; *p* = 0.002). The heterogeneity was low (*I*^2^ = 24%) and analyzed in FEM (**Figure 3A**).

#### Clavien–Dindo Grade ≥III

The Clavien–Dindo classifications of complications were referred in four studies (14, 18, 22, 23). These four studies had no heterogeneity (*I*^2^ = 0%), and therefore, the FEM was used. The results showed that Clavien–Dindo grade ≥III was lower in the LPD group (OR: 0.61; 95% CI 0.40 to 0.91; *p* = 0.02) (**Figure 3B**).

#### Thirty-Day Mortality

Six studies (14–16, 19–21) that included 10,884 patients (1,272 and 9,612 underwent LPD and OPD, respectively) assessed the 30-day mortality. The *Q* test showed six studies with low heterogeneity (*I*^2^ = 0%), and therefore, the FEM was adopted. The pooled data showed no difference in 30-day mortality (OR: 1.02; 95% CI 0.72 to1.44; *p* = 0.93) (**Figure 3C**).

#### Postoperative Pancreatic Fistula

**Figure 4:**
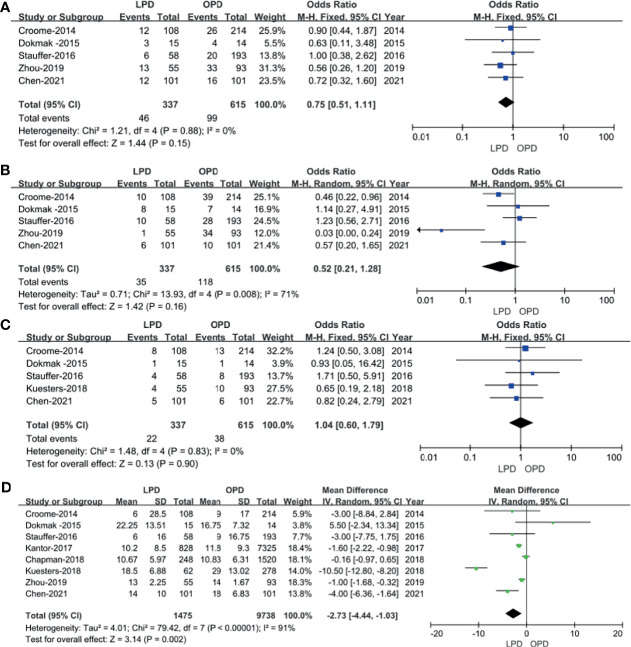
Forest plot of the comparison of LPD *versus* OPD for overall complication rates. **(A)** Forest plot for postoperative pancreatic fistula; **(B)** forest plot for delayed gastric emptying; **(C)** forest plot for postpancreatectomy hemorrhage; **(D)** forest plot for length of stay.

Postoperative pancreatic fistula (POPF) incidence rates were described for 952 patients in five studies (14, 16, 18, 22, 23). No significant differences in POPF rates were observed between these two groups (OR: 0.75; 95% CI 0.51 to 1.11; *p* = 0.15), with a low heterogeneity (*I*^2^ = 0%) in FEM (**Figure 4A**).

#### Delayed Gastric Emptying

Five studies (14, 16, 18, 22, 23) that encompassed 952 patients (337 underwent LPD and 615 underwent OPD) reported delayed gastric emptying rate, and the result of the meta-analysis indicated no difference in delayed gastric emptying (DGE) (OR: 0.52; 95% CI 0.21 to 1.28; *p* = 0.16), with a moderate heterogeneity (*I*^2^ = 71%) in REM (**Figure 4B**).

#### Postpancreatectomy Hemorrhage

Pooling the data of five studies (14, 16, 18, 20, 23) with 952 patients (337 underwent LPD and 615 underwent OPD) assessed postpancreatectomy hemorrhage, and the present analysis revealed no difference in postpancreatectomy hemorrhage (PPH) (WMD: 1.04; 95% CI 0.60 to 1.79; *p* = 0.90), with a low heterogeneity (*I*^2^ = 0%) in the FEM (**Figure 4C**).

#### Length of Stay

Eight studies (14, 16, 18–23) with a total of 11,213 patients (1,475 underwent LPD and 9,738 underwent OPD) investigated the LOS. The meta-analysis suggested a shorter LOS in the LPD group (MD = −2.73; 95% CI −4.44 to −1.03; *p* = 0.002), with high heterogeneity (*I*^2^ = 91%) in the FEM (**Figure 4D**).

### Short−Term Oncological Outcomes

#### R0 Resection Rate

**Figure 5:**
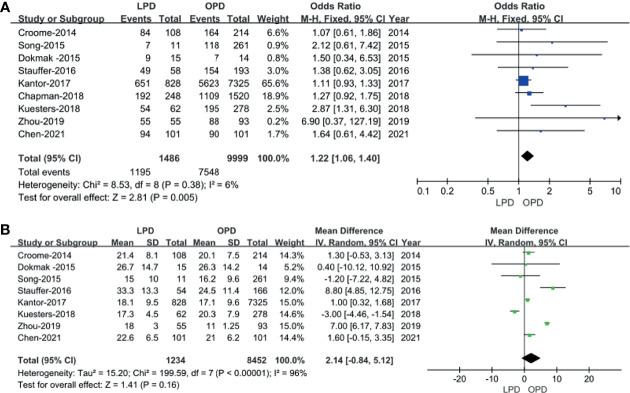
Forest plot of the comparison of LPD *versus* OPD for short−term oncological outcomes. **(A)** Forest plot for R0 resection rate; **(B)** forest plot for lymph node dissection.

In total, 9 studies including 11,194 patients (1,195 underwent LPD and 9,999 underwent OPD) provided data regarding the R0 resection rate (14–16, 18–23). We found that LPD was associated with a higher R0 resection rate (OR: 1.22; 95% CI 1.06–1.40; *p* = 0.005), with low heterogeneity (*I*^2^ = 6%) as shown in the FEM (**Figure 5A**).

#### Lymph Node Dissection

Eight studies (14–16, 18–20, 22, 23) including 9,686 patients (1,234 underwent LPD and 8,452 underwent OPD) assessed the number of lymph node dissection. These eight studies had great heterogeneity (*I*^2^ = 96%), and therefore, the REM was used. The results revealed no difference in lymph node dissection (WMD: 2.14; 95% CI −0.84 to 5.12; *p* = 0.16) (**Figure 5B**).

### Long−Term Oncological Outcomes

#### Overall Survival

**Figure 6:**
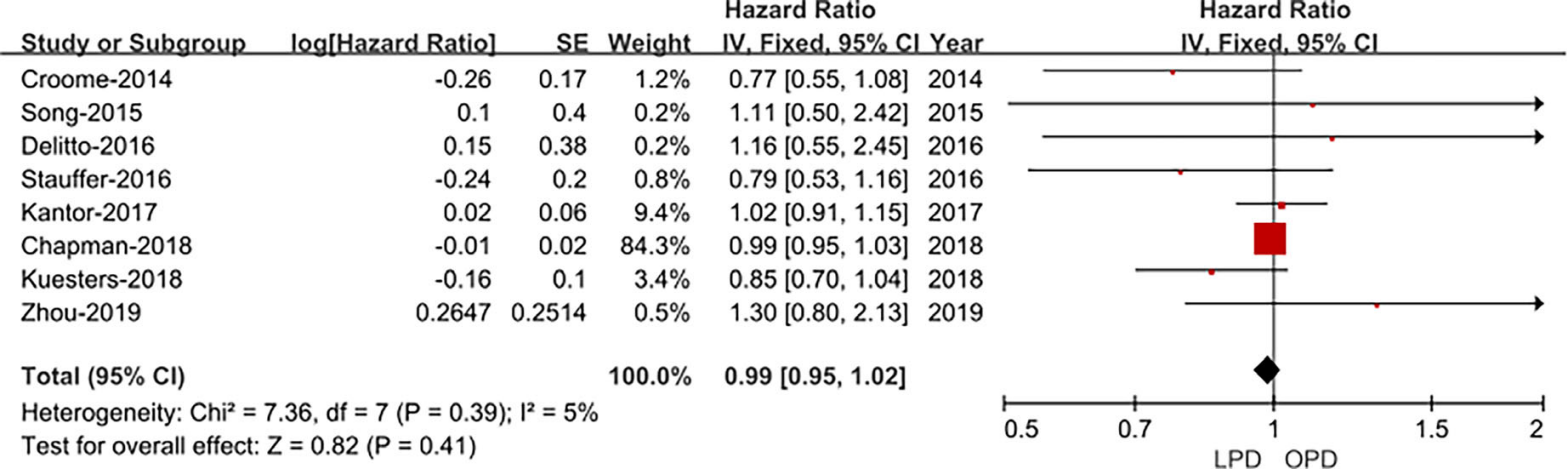
Forest plot of the comparison of LPD *versus* OPD for overall survival time.

Eight studies (14, 15, 17–22) assessed overall survival, and the result of the meta-analysis revealed LPD and OPD have similar overall survival time (HRs: 1.30; 95% CI 0.80 to 2.13; *p* = 0.41), with low heterogeneity (*I*^2^ = 5%) and analyzed in the FEM (**Figure 6**).

#### Long−Term Survival

**Figure 7:**
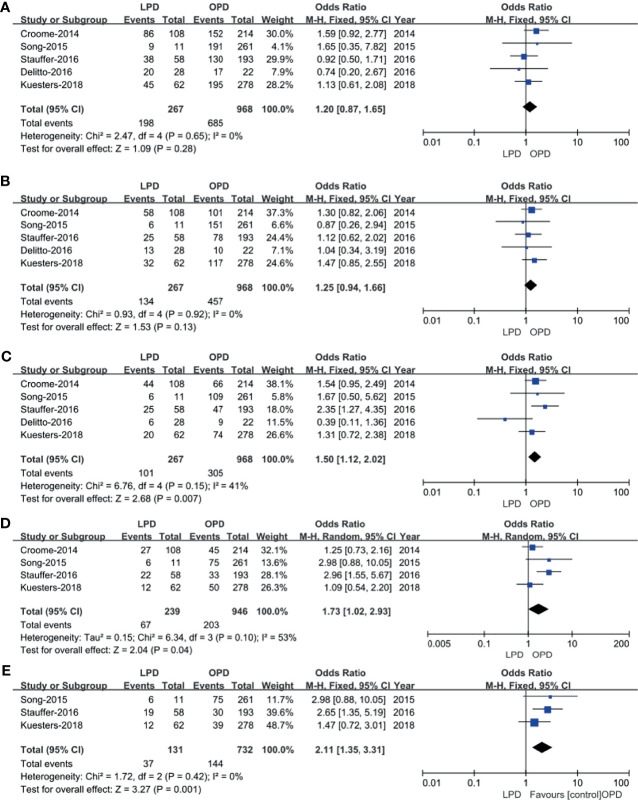
Forest plot of the comparison of LPD *versus* OPD for long−term oncological outcomes. **(A)** Forest plot for 1-year survival time; **(B)** forest plot for 2-year survival time; **(C)** forest plot 3-year survival time; **(D)** forest plot for 4-year survival time; **(E)** forest plot for 5-year survival time.

Five studies (14, 15, 17, 18, 20) that included 1,235 patients (267 underwent LPD and 968 underwent OPD) assessed 1-year survival time, 2-year survival time, and 3-year survival time, and the result of the meta-analysis showed no statistically significant difference in 1-year survival time and 2-year survival time between the two groups (**Figures 7A, B****)**. However, compared with the OPD group, the 3-year survival time was longer in the LPD group (37.8% *vs.* 31.5%, *p* = 0.007) (**Figure 7C**). Four studies (15, 16, 19, 21) that included 1,185 patients assessed 4-year survival, and three studies (16, 19, 21) that included 863 patients assessed 5-year survival, and the result of the meta-analysis showed LPD has longer 4-year survival and 5-year survival time (28.0% *vs.*. 21.5%, *p* = 0.04; 28.2% *vs.* 19.7%, *p* = 0.001) (**Figures 7D, E****)**.

#### Time to Starting Adjuvant Chemotherapy After Surgery

**Figure 8:**
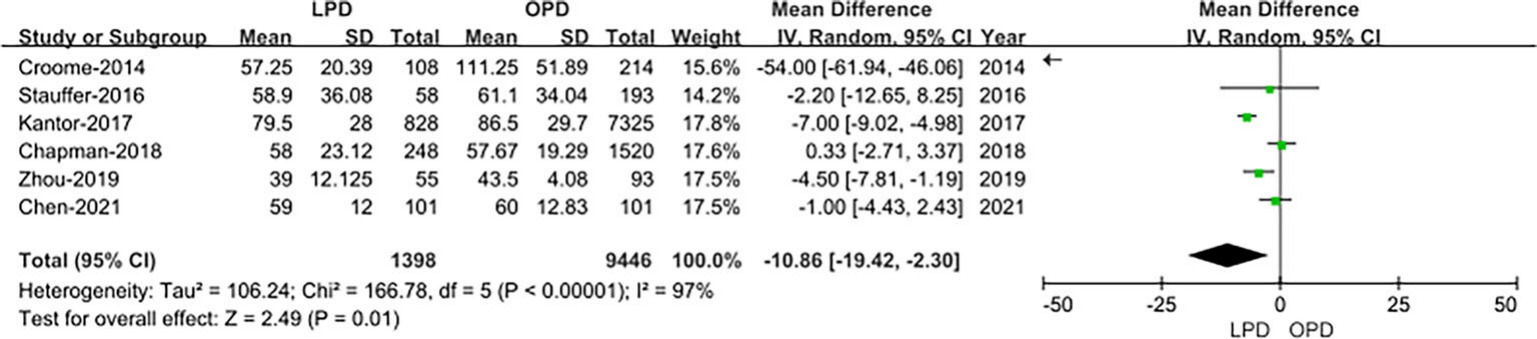
Forest plot of the comparison of LPD *versus* OPD for time to starting adjuvant chemotherapy after surgery.

The data of time to starting adjuvant chemotherapy after surgery were available in six studies (14, 18, 19, 21–23). The result of the meta-analysis revealed time to starting adjuvant chemotherapy after surgery was earlier in the LPD group (OR: −10.86; 95% CI −19.42 to −2.30; *p* = 0.01). Heterogeneity was high (*I*^2^ = 97%) and analyzed in REM (**Figure 8**).

#### Publication Bias

**Figure 9:**
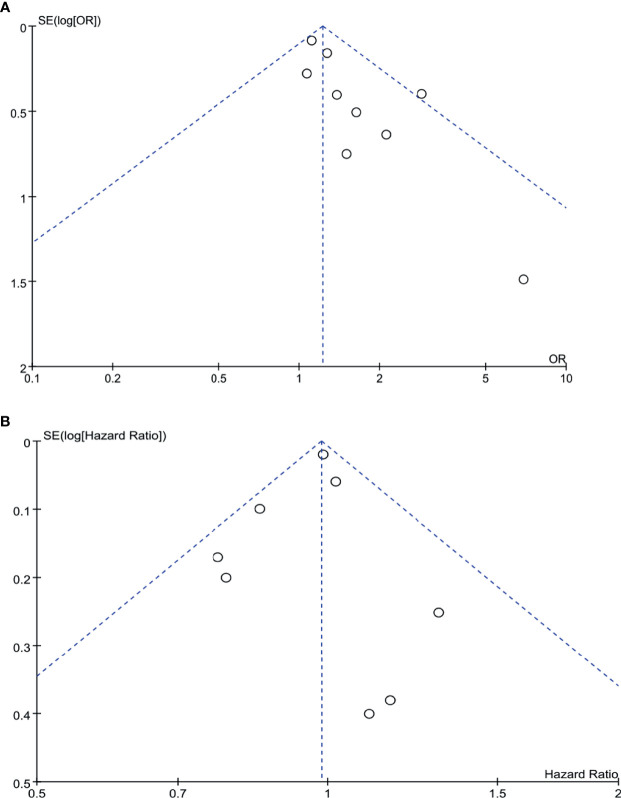
Funnel plots for R0 rate **(A)** and overall survival **(B)**.

Begg’s funnel plot was drawn for each outcome and adopted to investigate publication bias. All studies lie inside the 95% CIs in the funnel plot of R0 rate and overall survival, which indicated no obvious publication bias (**Figure 9**).

## Discussion

Since 1994, the first case of LPD was performed by Gagner et al., and the magnifying effect and visual field advantage of laparoscopic technique made it popular. Almost a decade later, Giulianotti et al. performed the first robotic pancreaticoduodenectomy (RPD) in Italy in 2003. RPD and LPD are both minimally invasive treatments for pancreatic and periampullary malignancies and some benign diseases. At present, with the development of laparoscopic surgical instruments and the accumulation of surgical experience, LPD has been proven to be safe and feasible and has many advantages over OPD, but the operation is more difficult and time-consuming (24, 25). Owing to the complicated operation process and high requirements for surgeons, LPD is considered as the “Everest” of abdominal minimally invasive surgery. Many institutions choose to include patients with benign diseases when they carry out LPD in the initial stage. In order to compare the real difference between LPD and OPD in the treatment of PDAC, we analyzed the data from the literature that cases were pathologically diagnosed as PDAC. Finally, the present meta-analysis included the latest studies from 2014 to 2021 to compare the safety and efficacy of LPD and OPD for PDAC. Although none of the studies was RCTs, most of included studies were relatively high quality according to NOS. Finally, our study included 10 publications with 11,535 (LPD 1,514 *vs.* OPD 10,021) patients and reflects the newest surgical results for the treatment of PDAC.

Three previous meta-analyses comparing perioperative and oncologic outcomes of LPD to OPD were published in the last 3 years. However, two of them (7, 9) included an article about RPD in the treatment of PDAC. The study of Chen et al. (7) is the only meta-analysis that focuses on LPD and OPD. Six articles were included in the study of Chen et al. and focused on oncologic outcomes and long-term survival. They found that LPD was associated with longer 3-year survival, 4-year survival, and 5-year survival time, and there was no difference in 1-year survival, 2-year survival time, lymph nodes harvested, the number of positive lymph nodes, and R0 rate. The meta-analysis of Yin et al. (9) covered 9,144 PDAC participants from six retrospective studies, and the meta-analysis of Sun et al. (8) covered 1,377 minimally invasive pancreaticoduodenectomy (LPD and RPD) and 9,865 OPD from nine retrospective studies. They advised that LPD was associated with higher R0 rate, longer operative time, fewer blood loss, lower transfusion rate, and shorter length of hospital stay. There were no significant differences in morbidity, POPF, DGE, the number of harvested lymph nodes, and mortality.

Compared with the results of other studies, our study included some recent studies and excluded the study comparing RPD and OPD. The results of our meta-analysis showed LPD has a shorter LOS and less blood loss but no difference in blood transfusion. Why do we have different results for blood loss and the same blood transfusion rate in the two groups? It is because five studies with a total of 1,195 patients (14, 15, 18, 22, 23) had reported blood loss. Another five studies (14, 18, 20, 22, 23) with 1,260 patients reported blood transfusion, and heterogeneity was very high (*I*^2^ = 81%), indicating that there is obvious publication bias. The results of our meta-analysis showed LPD has a longer operative time than OPD, which was similar with the study of Yin et al. The main factors that lead to the longer operation time of LPD are longer pancreatectomy and digestive tract reconstruction under a laparoscope.

The current meta-analysis shows non-significant difference in the 30-day mortality, overall complication rates, POPF, and the incidence of severe complications (Clavien–Dindo 3/4 grade complications) between the two groups, indicating that the safety of the two groups was similar. PPH is one of the most severe complications after pancreatic surgery. The meta-analysis of Floortje van Oosten et al. (26) showed that the incidence of PPH was about 5% after pancreatectomy, and the overall mortality caused by PPH accounted for 21%. Our meta-analysis shows that the rate of PPH was 6% (60/952 patients; range 4.1%–10.7%), and pooling the data of five studies (14, 16, 18, 20, 23) with 952 patients revealed no difference in PPH between the LPD and OPD groups.

Negative margin and the number of lymph node dissection are two important malignancy prognosis factors in PD. Pooled data from this meta-analysis revealed that LPD has a higher rate of R0 resection than OPD. We think that this may be explained by the fact that patients with PDAC in early stage were selected to perform LPD. From the perspective of tumor radical effect, the results of this study show that the two surgical methods have the same effect in the number of lymph node dissection, suggesting that LPD and OPD have the same tumor radical effect, which is basically consistent with the results of most existing clinical studies.

When it comes to long-term survival, according to our search, there are still no RCTs comparing the long-term survival between LPD to OPD in patients with PDAC. At present, the largest overall survival outcomes data of LPD in the treatment of PDAC come from the National Cancer Database (NCDB). Kantor et al. reported that 8,213 patients with PDAC underwent PD (828 underwent LPD and 7,385 underwent OPD) and revealed a non-significant difference in survival time in the two groups (20.7 *vs.* 20.9 months) (19). However, Chapman et al. compared the survival data of 1,768 patients with PDAC (248 underwent LPD and 1,520 underwent OPD) also from NCDB and suggested that LPD and OPD can achieve a median overall survival of 19.8 and 15.6 months, respectively (*p* = 0.022) (21). Although our meta-analysis revealed that the LPD group has an earlier time to get starting adjuvant chemotherapy after surgery, there is no significant difference in OS (HRs: 1.30; 95% CI 0.80 to 2.13; *p* = 0.41). In some ways, the pooled data demonstrated that LPD is not oncologically inferior to OPD and even can achieve superior oncologic outcome compared with OPD.

To evaluate the safety and efficiency of LPD for PDAC, this meta-analysis included 10 studies and revealed that LPD was comparable to OPD. But some limitations in this study should be considered. First, no RCTs were included which may contribute selection bias. Furthermore, of the 10 included studies, TNM stage, tumor size, and differentiation degree of patients with PDAC have not been reported in some studies. What is more, only a few studies reported long-term survival outcomes such as overall survival and 5-year survival time of LPD. Therefore, further large-scale prospective comparative studies and RCTs are expected to provide more convincing results to further assess the effectiveness and safety of LPD for patients with PDAC.

## Conclusion

In summary, the present meta-analysis revealed that LPD is a technically and oncologically safe and feasible approach for PDAC patients and provided similar long-term overall survival time with OPD.

## Data Availability Statement

The raw data supporting the conclusions of this article will be made available by the authors, without undue reservation.

## Author Contributions

Study concept and design (QF, KY), acquisition of data (All authors), analysis and interpretation of data (QF, ZX, WL), drafting of the manuscript (QF, JD, WL), critical revision of the manuscript for important intellectual content (KY), administrative, technical, or material support, study supervision (YZ). All authors contributed to the article and approved the submitted version.

## Funding

This work was supported by grants from the National Key Technologies R&D Program (2018YFC1106800) and the Natural Science Foundation of China (82173124, 82173248, 82103533, 82002572, 82002967, 81972747, and 81872004).

## Conflict of Interest

The authors declare that the research was conducted in the absence of any commercial or financial relationships that could be construed as a potential conflict of interest.

## Publisher’s Note

All claims expressed in this article are solely those of the authors and do not necessarily represent those of their affiliated organizations, or those of the publisher, the editors and the reviewers. Any product that may be evaluated in this article, or claim that may be made by its manufacturer, is not guaranteed or endorsed by the publisher.
